# Incidence of Epileptiform EEG Activity in Children during Mask Induction of Anaesthesia with Brief Administration of 8% Sevoflurane

**DOI:** 10.1371/journal.pone.0040903

**Published:** 2012-07-19

**Authors:** Barbara Schultz, Christian Otto, Arthur Schultz, Wilhelm Alexander Osthaus, Terence Krauß, Thorben Dieck, Björn Sander, Niels Rahe-Meyer, Konstantinos Raymondos

**Affiliations:** Department of Anaesthesiology and Intensive Care Medicine, Hannover Medical School, Hannover, Germany; University of Colorado, United States of America

## Abstract

**Background:**

A high incidence of epileptiform activity in the electroencephalogram (EEG) was reported in children undergoing mask induction of anaesthesia with administration of high doses of sevoflurane for 5 minutes and longer. This study was performed to investigate whether reducing the time of exposure to a high inhaled sevoflurane concentration would affect the incidence of epileptiform EEG activity. It was hypothesized that no epileptiform activity would occur, when the inhaled sevoflurane concentration would be reduced from 8% to 4% immediately after the loss of consciousness.

**Methodology/Principal Findings:**

70 children (age 7–96 months, ASA I–II, premedication with midazolam) were anaesthetized with 8% sevoflurane in 100% oxygen via face mask. Immediately after loss of consciousness, the sevoflurane concentration was reduced to 4%. EEGs were recorded continuously and were later analyzed visually with regard to epileptiform EEG patterns. Sevoflurane at a concentration of 8% was given for 1.2±0.4 min (mean ± SD). In 14 children (20%) epileptiform EEG patterns without motor manifestations were observed (delta with spikes (DSP), rhythmic polyspikes (PSR), epileptiform discharges (PED) in 10, 10, 4 children (14%, 14%, 6%)). 38 children (54%) had slow, rhythmic delta waves with high amplitudes (DS) appearing on average before DSP.

**Conclusions/Significance:**

The hypothesis that no epileptiform potentials would occur during induction of anaesthesia with a reduction of the inspired sevoflurane concentration from 8% to 4% directly after LOC was not proved. Even if 8% sevoflurane is administered only briefly for induction of anaesthesia, epileptiform EEG activity may be observed in children despite premedication with midazolam.

## Introduction

The volatile anaesthetic sevoflurane is in widespread use for mask induction of anaesthesia in children. It is characterized by lack of pungency and upper airway irritation and a favourable cardiovascular safety profile [1]. The use of high concentrations has been recommended to accelerate the loss of consciousness [2], [3]. However, seizure-like motor activity [4]–[6] as well as electroencephalographic abnormalities [7]–[8] have been reported in children anaesthetized with sevoflurane.

Several risk factors for the occurrence of epileptiform electroencephalogram activity during anaesthesia in adults and in children have been proposed. Among these factors are the speed of induction of anaesthesia [9], a high alveolar concentration of sevoflurane [8], [10], [11], hyperventilation [12], and female gender [13].

The aim of this study was to investigate during induction of anaesthesia in children whether an early reduction of the sevoflurane concentration from 8% to 4% immediately after the loss of consciousness (LOC) affects the incidence of epileptiform EEG activity. We hypothesized that no epileptiform potentials would occur. EEGs with sharp transients occur typically when high endtidal sevoflurane concentrations are present [10]. By minimizing the application time of 8% sevoflurane, higher endtidal sevoflurane concentrations should be avoided.

Depending on the presence of epileptiform activity, it was a further aim to characterize the observed epileptiform EEG patterns.

## Materials and Methods

### Ethics Statement

EEGs were analyzed as part of a prospective study that was carried out to determine norm values of monitoring parameters in children during routinely performed anaesthesia. The study had been approved by the ethics committee of Hannover Medical School. In accordance with the ethics committee statement, written informed consent for anaesthesia and surgery was obtained from the parents.

When epileptiform EEG activity was observed during the analysis of the data, the EEG patterns were analyzed.

**Figure 1:**
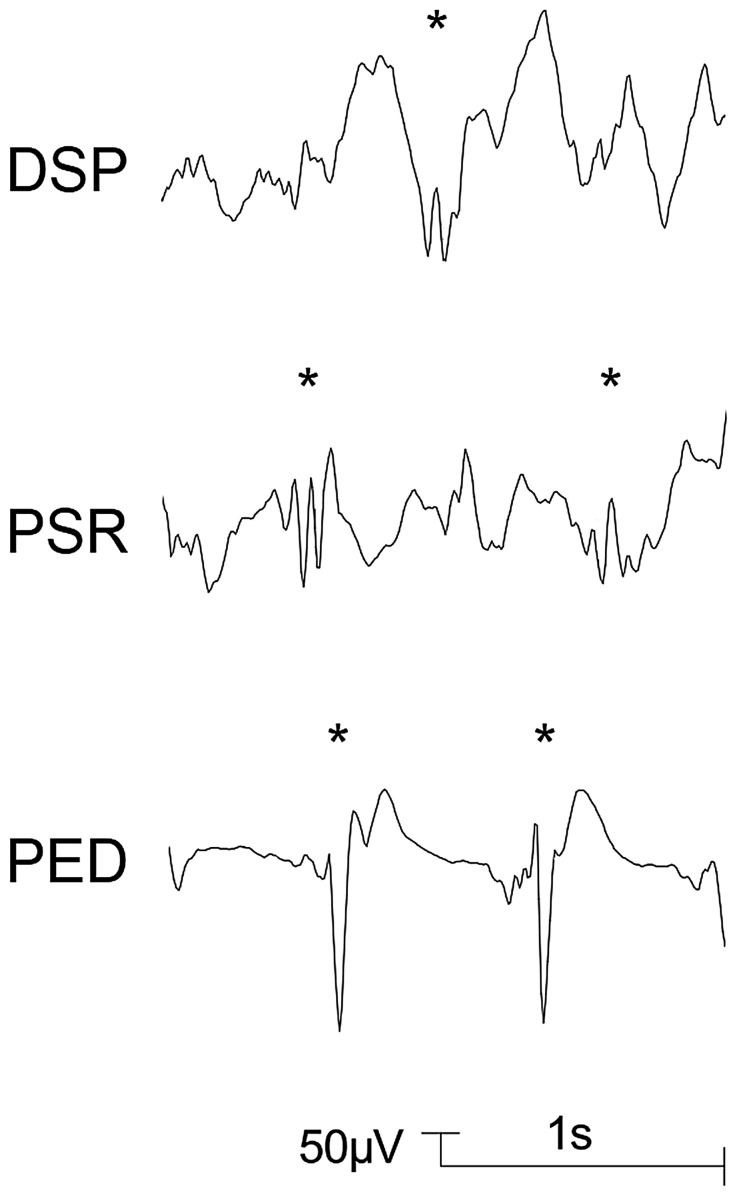
Delta with spikes (DSP), rhythmic polyspikes (PSR), and periodic epileptiform discharges (PED). Typical examples are indicated by asterisks.

### Patients and Study Design

70 children aged 7 months to 8 years with American Society of Anesthesiologists (ASA) physical status I–II were included in this analysis. All patients were scheduled for elective ear, nose and throat surgery.

Severe cardiovascular or respiratory diseases and contraindications against any of the drugs used were exclusion criteria. Patients with neurological diseases were not generally excluded, but a history of seizure disorder was an exclusion criterion. Four of the EEGs included in this evaluation were recorded in children with a history of or with current neurologic pathology: one child with a microcephalus, another with a hydrocephalic shunt, one child with periventricular leucomalacia and one child who had had a cerebral abscess. These children would have received mask induction with sevoflurane in normal clinical practice, and our aim was to analyze the incidence of epileptiform EEG activity under conditions as close to normal as possible.

As oral premedication, 68 children received 0.5 mg/kg body weight midazolam in water (maximum dose 10 mg) 45 minutes before anaesthesia. Two children refused to take the premedication.

Before induction of anaesthesia, standard monitoring including non-invasive blood pressure measurement, pulse oximetry, electrocardiogram (ECG) and electroencephalogram (EEG) was established and the anaesthetic circuit was prefilled for 5 min with 8% sevoflurane.

**Figure 2:**
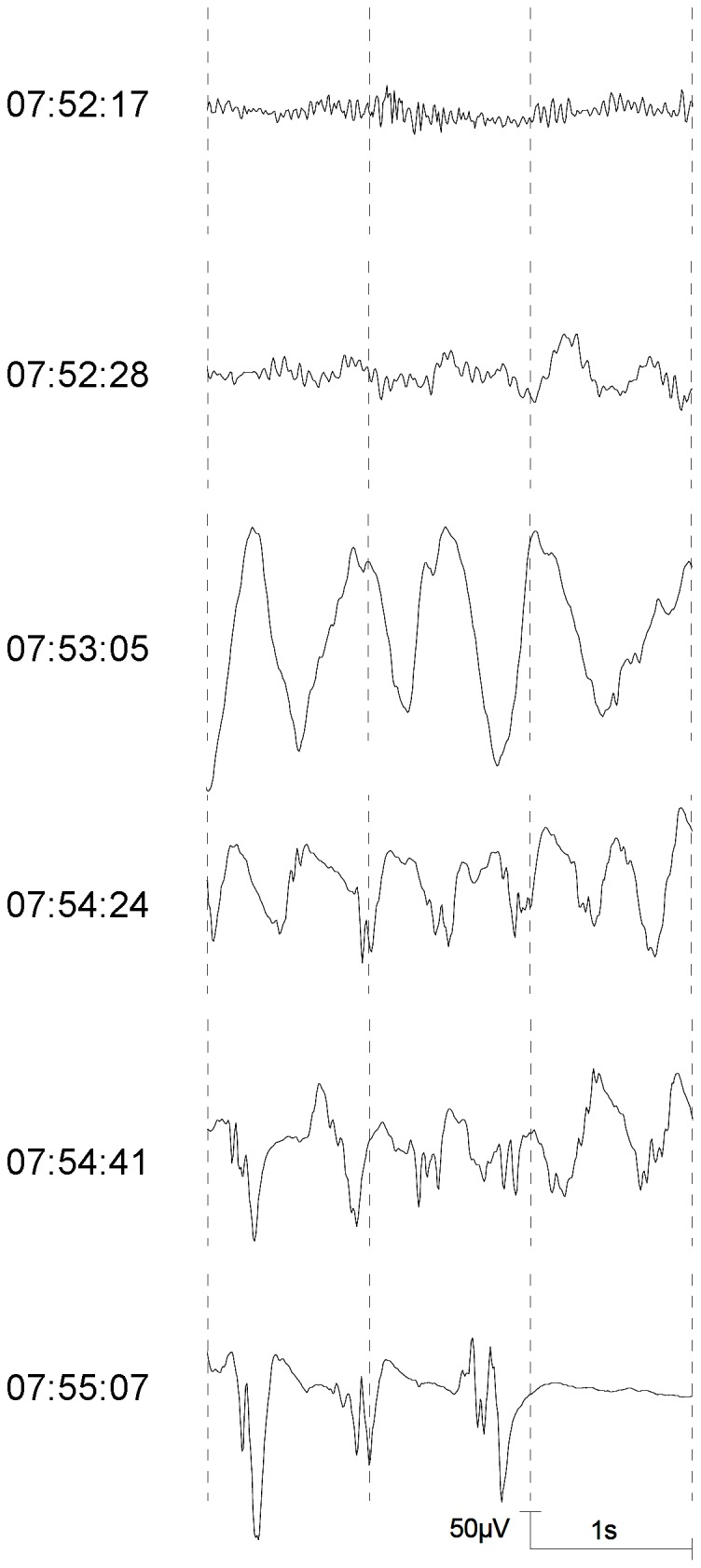
High amplitude, regular delta activity (07:53:05) and epileptiform potentials (from 07:54:24 on) during an induction of anaesthesia.

**Figure 3:**
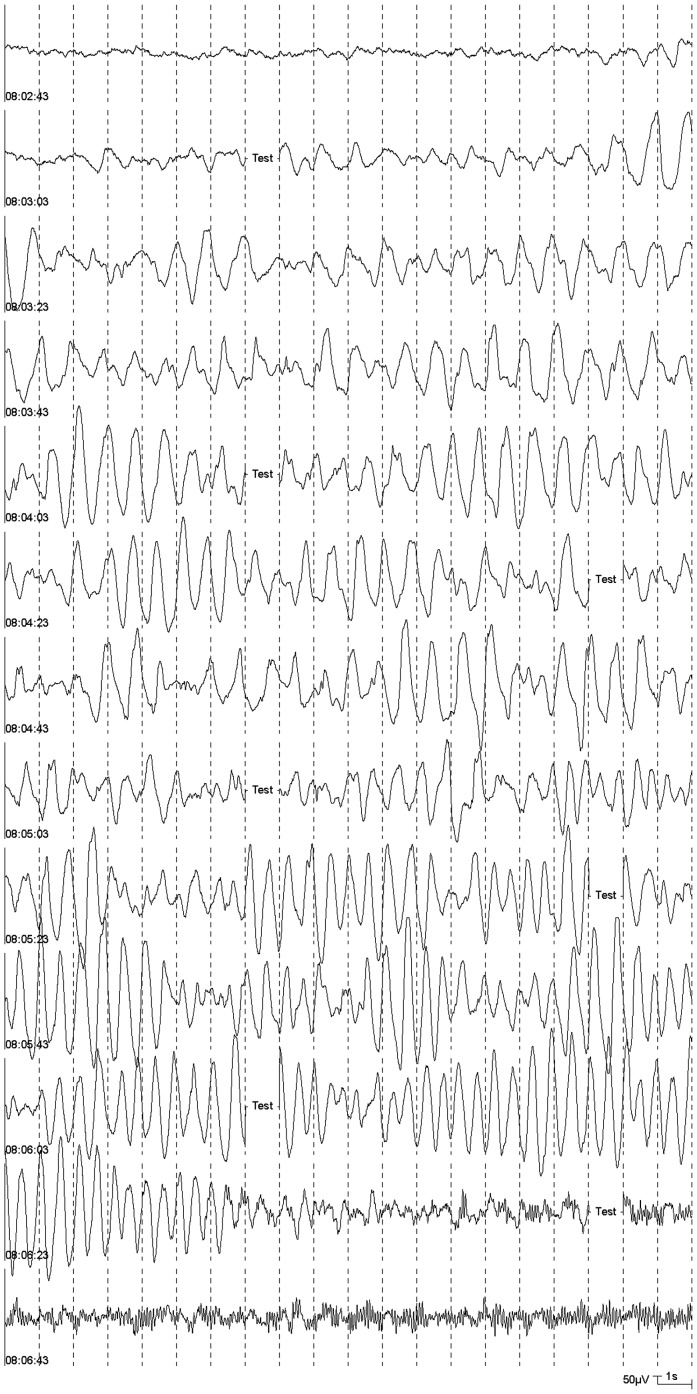
Spindle-shaped, regular delta activity with high amplitudes (DS) starting at 08:03:21.

**Table 1:** Demographic data.

	Without epileptiform potentials	With epileptiform potentials	p
Female/Male	27/29	6/8	0.7195
Age (months)	47.0±24.9	46.0±24.3	0.8920
Weight (kg)	16.2±7.6	16.7±6.6	0.8186
Height (cm)	100.4±18.0	101.1±18.1	0.8976

**Table 2:** Duration of sevoflurane administration, endtidal sevoflurane concentrations, and haemodynamic parameters (mean ± standard deviation).

	Without epileptiformpotentials	With epileptiform potentials	p
Duration of 8% sevoflurane administration (s)	72.8±25.9	76.2±25.0	0.6535
Sevoflurane concentration (exsp.) before reduction to 4 Vol.-% (Vol.-%)	5.1±1.1	4.7±1.1	0.2300
Sevoflurane concentration (exsp.) before propofol bolus (Vol.-%)	3.2±0.6	3.4±0.8	0.2501
Heart rate before induction (beats/min)	114.4±24.7	114.5±25.1	0.9885
Blood pressure (sys.) before induction (mmHg)	103.0±13.4	105.6±13.9	0.5229
Blood pressure (dia.) before induction (mmHg)	61.6±11.5	64.1±14.5	0.4849
Heart rate before propofol bolus (beats/min)	117.8±23.8	126.2±21.4	0.2488
Blood pressure (sys.) before propofol bolus (mmHg)	95.0±14.8	96.7±16.4	0.7111
Blood pressure (dia.) before propofol bolus (mmHg)	51.5±10.3	52.5±9.2	0.7674

**Table 3:** Visually classified EEG patterns (Median [Min–Max]).

EEG pattern	n	Time to onset (s)	Number of seconds occupied by pattern
Slow delta (DS)	38	**78.5** [17–171]	**25.5** [3–190]
Delta with spikes (DSP)	9	**127** [53–276]	**17** [6–37]
Rhythmic polyspikes (PSR)	10	**144** [95–350]	**23** [5–118]
Periodic epileptiform discharges (PED)	4	**230** [127–410]	**12.5** [4–30]

Sevoflurane mask induction was performed in all children with 8% sevoflurane in oxygen and a fresh gas flow of 8 l/min using a Rendell-Baker mask. Loss of consciousness (LOC) was defined as loss of the eyelash reflex and absence of spontaneous movements. Immediately after LOC, the inspired sevoflurane concentration was reduced to 4%. The patients breathed spontaneously or ventilation was assisted manually to achieve normoventilation (endtidal CO_2_ 35–40 mmHg). An intravenous line was placed and then propofol, remifentanil and mivacurium were given for tracheal intubation.

This way of anaesthesia induction in children was an established standard procedure in the department.

During the observation period, systolic and diastolic blood pressure, heart rate, and endtidal sevoflurane concentrations were noted and also the time of loss of consciousness.

### EEG Monitoring

The EEGs were recorded with the EEG monitor Narcotrend® (MT MonitorTechnik, Bad Bramstedt, Germany) [14]. After skin preparation with alcohol and abrasive paste, two standard ECG electrodes were positioned on the patient’s left and right forehead at maximum achievable distance and a third electrode as reference in the middle. The electrode impedances were below 6000 ohms.

### Visual EEG Analysis

The raw EEGs of all 70 patients were assessed visually from the beginning of sevoflurane inhalation to the start of the intravenous drug injection for tracheal intubation. The investigator (B. S.) who analyzed the EEGs offline is experienced in EEG interpretation and holds the EEG certificate from the German Society of Clinical Neurophysiology and Functional Imaging. She was blinded to the inspired and endtidal sevoflurane concentrations.

Patterns for the classification of EEGs during sevoflurane anaesthesia in children were proposed by Vakkuri et al. (2001) [8]. Based on this proposal, the following EEG patterns which were regarded as epileptiform patterns [8], [13] were distinguished in this study: DSP (delta with spikes; delta acitivity of any frequency with regular or irregular spikes), PSR (rhythmic polyspikes; waveform with more than two negative and positive deflections appearing at regular intervals, associated with slow wave or mixed frequency EEG activity between spike complexes) and PED (periodic epileptiform discharges) [8]. Examples for DSP, PSR, and PED are presented in Figure 1. A further pattern with spikes, SSP (suppression with spikes), that may appear during sevoflurane anaesthesia in children [8], was not observed in the analyzed EEG segments. A pattern without spikes but with noticeable rhythmicity, DS (slow delta; regular, rhythmic delta activity <2.0 Hz with high amplitudes) was included. This pattern is shown in Figures 2 and 3.

### Statistical Analysis

70 patients were included in the study. A power analysis showed, that this number of patients allows to achieve a significant result with a power of 0.9, if the portion of patients with epileptiform activity is 0.02 and the assumption of the null hypothesis is 0.00.

For the comparison of means the t-test was used, frequencies were evaluated with the Chi-square test (SAS, Version 9.2 (SAS Institute, Cary, USA)). Statistical significance was assumed for p<0.05.

## Results

The demographic data of the patients with and without epileptiform potentials are presented in Table 1. No significant differences between the two groups were found with regard to age, gender, weight, and height.

The duration of sevoflurane administration, the endtidal sevoflurane concentration at loss of consciousness, and the endtidal sevoflurane concentration before the injection of the propofol bolus did not differ significantly between the two groups (Table 2).

The EEG patterns DSP, PSR and PED were observed in 14 patients (20%): 10 patients had delta with spikes, 10 patients had rhythmic polyspikes, and 4 patients had periodic epileptiform discharges (Table 3). The four children with a history of or with current neurological pathology were not among these 14 patients.

The epileptiform patterns appeared in a typical sequence (Figure 2). Table 3 shows that the median time to the onset of DSP was shortest, followed by PSR and PED.

The pattern DS was found in the EEGs of 38 patients (Table 3, Figure 3). In 25 patients, this pattern was spindle-shaped with increasing and decreasing amplitudes. The highest amplitudes reached more than 1000 µV.

The haemodynamic parameters did not differ significantly between the two groups (Table 2).

Because of a prolonged induction time, the data of one patient were not included in the calculation of the time to the onset of DSP, the median number of seconds with DSP, the mean time between start of sevoflurane and first appearance of epileptiform potentials, and in the calculation of heart rate and blood pressure data noted directly before administration of the propofol bolus.

Mask induction was tolerated by all patients. Two children developed mild laryngeal spasms and no seizure-like movements were observed during the period under consideration.

## Discussion

Contrary to the hypothesis that epileptiform potentials would be avoided, epileptiform EEG patterns were observed in 20% of the patients of the study population although the administration times of 8% sevoflurane were relatively short. Because no other drug, except of oral midazolam for premedication, was given, this finding can be attributed to the use of sevoflurane for induction of anaesthesia.

Sevoflurane at a concentration of 8% was also used in studies by Vakkuri et al. [8] and Sonkajärvi et al. [15] in children. While in our study 8% sevoflurane (in 100% O_2_) was given for 73±26 s (1.2±0.4 min) on average, in these studies the administration times were considerably longer. Vakkuri et al. [8] gave 8% sevoflurane (in N_2_O/O_2_ 2:1) until 5 min after LOC and then, after intubation, 2% sevoflurane for 3 min. 80% of their patients, who after breathing spontaneously received slow assisted ventilation, had DSP, 20% had PSR. In the study by Sonkajärvi et al. [15], during administration of 8% sevoflurane (in N_2_O/O_2_ 1∶1) for 5 to 8 min and then 2% for 3 min, 19 of 20 normoventilated children developed multifocal spikes with a maximum over the frontal lobes. In both studies no motor phenomena were observed.

In the studies of Vakkuri et al. [8] and Sonkajärvi et al. [15] N_2_O was used, whereas in our the study no N_2_O was given. Kurita et al. [16] investigated the effect of 50% N_2_O on the frequency and extent of spike activities on electrocorticogram in epileptic patients under anaesthesia with 1.5 MAC sevoflurane and a small dose of fentanyl. The authors demonstrated that the induction of nitrous oxide significantly decreased the spike activity. If N_2_O had been given in our study, the number of patients with epileptiform potentials would possibly have been smaller.

Midazolam was given in similar doses for premedication by Vakkuri et al. [8], Sonkajärvi et al. [16] and in our study (Vakkuri et al.: 0.5 mg/kg, maximum dose 15 mg, Sonkajärvi et al.: 0.4 mg/kg, maximum dose 10 mg, own study: 0.5 mg/kg, maximum dose 10 mg). It has a significant anticonvulsant effect and, accordingly, may have influenced the results of all three studies similarly [17].

Niemienen et al. [17] did not observe epileptiform patterns during maintenance of anaesthesia with only 2% sevoflurane in children. In their study, midazolam was given for premedication and anaesthesia was induced with thiopental. The authors discuss the two possibilities that the lack of epileptiform potentials was due to the fact that midazolam and thiopental were given or to the concentration of sevoflurane that was used. But epileptiform patterns were observed in the studies of Vakkuri et al. [8] and Sonkajärvi et al. [15] in spite of the premedication with midazolam. In our own study, the two children without premedication did not develop epileptiform potentials.

The patients in the studies by Vakkuri et al. [8] and Sonkajärvi et al. [16] were older than the patients in our study (Vakkuri et al.: 6±2 years, Sonkajärvi et al.: 6.9±1.9 years), but no age effect concerning the occurrence of epileptiform potentials was found in our study. When comparing the results of the three studies, it must be taken into consideration that there are differences in anaesthetic technique, furthermore, the visual EEG interpretation was performed by different persons.

The endtidal sevoflurane concentrations at the moment of switching from 8% to 4% sevoflurane were not significantly different in the groups of patients without and with epileptiform potentials in our study (5.1±1.1 vs. 4.7±1.1 Vol.-%). The first epileptiform potentials appeared 134.5±58.5 s after the start of the sevoflurane administration, i. e. about 1 min after LOC and the subsequent reduction of the inspired sevoflurane concentration. Only in 2 of 13 patients, the epileptiform potentials began later than 2 min after the change of the sevoflurane vaporizer setting to 4%. Reports from the literature show that the incidence and the periodicy of epileptiform EEG changes correlate with an increasing expired fraction of sevoflurane [18]. In our study, the administration time of sevoflurane at high concentrations and consequently the time that was available for equilibration between the different body compartments, including the brain, was comparably short, providing an explanation for the relatively low incidence of epileptiform potentials.

Julliac et al. [13] described that women had a higher risk to develop epileptiform patterns during sevoflurane induction than men. But gender did not have a significant influence on the occurrence of epileptiform patterns in children in our study.

No relationship was found between the incidence of epileptiform potentials and the children’s age. 5 of the 70 children were younger than 1 year, 2 of these had epileptiform potentials in their EEGs. In the children of this study, who were between 7 months and 8 years old, EEG changes typical for induction of anaesthesia with sevoflurane could be seen. Therefore no age group was excluded from the analysis.

The clinical significance of the epileptiform EEG patterns seen during sevoflurane anaesthesa is still not clear [15].

According to Jöhr and Berger [1], sevoflurane has become the most popular agent for inhalation induction in paediatric patients in the developed world. As one main drawback of sevoflurane, the authors name uncertainties concerning the relevance of its EEG stimulating effects.

The evidence of seizure activity in EEG recordings during higher concentrations of sevoflurane was the reason for Holzki and Kretz [19] to consider the use of sevoflurane in concentrations not higher than 5.0–5.5% in N_2_O for induction of anaesthesia. Constant et al. [18] remarked that the wide use of cerebral function monitoring may permit optimization of sevoflurane dose, and avoidance of burst suppression pattern and major epileptiform signs in fragile subjects, notably the very young and the very old. Voss et al. [20] also recommended that EEG monitoring should be considered when sevoflurane is used.

Our hypothesis that no epileptiform potentials would occur during induction of anaesthesia with a reduction of the inspired sevoflurane concentration from 8% to 4% directly after LOC was not proved. But with the induction technique used in this study, a smaller portion of patients developed epileptiform patterns than in other studies with longer administration times of 8% sevoflurane. EEG monitoring can be used to identify those patients who still develop epileptiform patterns and to adjust the sevoflurane dose accordingly.
